# Binary Sensors-Based Privacy-Preserved Activity Recognition of Elderly Living Alone Using an RNN

**DOI:** 10.3390/s21165371

**Published:** 2021-08-09

**Authors:** Tan-Hsu Tan, Luubaatar Badarch, Wei-Xiang Zeng, Munkhjargal Gochoo, Fady S. Alnajjar, Jun-Wei Hsieh

**Affiliations:** 1 Department of Electrical Engineering, National Taipei University of Technology, Taipei 10617, Taiwan; thtan@ntut.edu.tw; 2 Department of Electronics, School of Information and Communication Technology, Mongolian University of Science and Technology, Ulaanbaatar 13341, Mongolia; luubaatar@must.edu.mn; 3 Wistron Corporation, Taipei 11469, Taiwan; bruce_zeng@wistron.com; 4 Department of Computer Science and Software Engineering, College of Information Technology, United Arab Emirates University, Al-Ain P.O. Box 15551, United Arab Emirates; fady.alnajjar@uaeu.ac.ae; 5 College of AI, National Chiao Tung University, Hsinchu 30010, Taiwan; jwhsieh@nctu.edu.tw

**Keywords:** privacy-preserving, device-free, long short-term memory, previous activity, *begin time-stamp*, convolutional neural network, infrared

## Abstract

The recent growth of the elderly population has led to the requirement for constant home monitoring as solitary living becomes popular. This protects older people who live alone from unwanted instances such as falling or deterioration caused by some diseases. However, although wearable devices and camera-based systems can provide relatively precise information about human motion, they invade the privacy of the elderly. One way to detect the abnormal behavior of elderly residents under the condition of maintaining privacy is to equip the resident’s house with an Internet of Things system based on a non-invasive binary motion sensor array. We propose to concatenate external features (*previous activity* and *begin time-stamp*) along with extracted features with a bi-directional long short-term memory (Bi-LSTM) neural network to recognize the activities of daily living with a higher accuracy. The concatenated features are classified by a fully connected neural network (FCNN). The proposed model was evaluated on open dataset from the Center for Advanced Studies in Adaptive Systems (CASAS) at Washington State University. The experimental results show that the proposed method outperformed state-of-the-art models with a margin of more than 6.25% of the *F*_1_ score on the same dataset.

## 1. Introduction

The world’s population has aged over the past few decades. In 2018, for the first time, there were more people aged 65 and over than those younger than five, and the elderly population is likely to have doubled by 2050 [1,2]. Moreover, in 2050, the 1.5 billion people older than 65 will outnumber those aged between 15 and 24. This dramatic increase in the elderly population is due to improved quality of life and better healthcare [3,4,5,6], especially the decrease in tobacco use in men and cardiovascular disease in recent decades [3]. Another important factor that affects the growth of the elderly population is the falling birth rate; the average number of live births per woman was only 2.5 worldwide in 2019 and is likely to decrease further [1]. Studies have shown that both high- and low-income countries are experiencing increased life expectancy [4,5].

Elderly people tend to live alone [7,8,9,10,11]. For example, in the United States of America, the percentage of elderly people living alone was 40% in 1990 and 36% in 2016 [12]. In the Republic of Korea, 22.8% of elderly people live alone, almost one in five [8]. One of the reasons is that some elderly people prefer to preserve their privacy [13,14]. However, elderly people living alone are more susceptible to loneliness, illness, and home accidents than those who live with a partner or family [9,15]. Early detection of illness and home accidents is crucial if solitarily-living elderly people are to receive timely and potentially life-saving help [16,17].

As the latest technological development, the Internet of Things (IoT) enables consumers and businesses to have versatile devices connected to the Internet [18,19,20,21,22]. In elderly care and monitoring systems, the use of the IoT is becoming prevalent [23,24,25,26], and monitoring the activities of daily living (ADLs) of elderly people is crucial in indicating their activity level [27].

Previous studies have proposed elderly-monitoring systems based on wearable devices [23,28,29,30,31,32] with the main function of classifying the ADLs of elderly people. However, some people are uncomfortable with wearable devices, and if they choose to wear one, then the favorite part of the body for wearing it is the wrist [33]. In addition to recognizing ADLs, crucial for monitoring elderly people living alone is detecting (i) abnormal activities such as falling [34,35], (ii) early signs of some diseases, and (iii) unusual instances for people with certain diseases [28,36,37]. However, although wearable devices provide accurate information about motion, they are inconvenient for daily use because of the problems such as the need to attach sensors to the body or skin, battery life expectancy, and the probability of abandonment in case of curiosity [38,39,40,41]. Although many different activity classification methods have been suggested for wearable devices, the recent prominence of machine learning (ML) has caused researchers to focus in particular on human activity recognition (HAR) models based on deep learning [38,42,43,44,45].

Camera-based monitoring systems [38,46,47,48,49] solve the problem of having an inconvenient wearable device attached to one’s body or skin. Although various HAR models have been suggested for recognizing ADLs, those based on ML are now playing a major role [22,48,49,50,51,52]. An example is HAR based on a dynamic Bayesian network for detecting the abnormal actions of elderly people from camera video [53]. However, although camera-based systems provide accurate information about human posture, privacy is a major concern [54,55,56]. Moreover, previous research [54] showed that elderly people tend to change their behavior once they are aware of the camera. To minimize the invasion of privacy associated with camera-based technology, low-resolution infrared or depth-camera systems have been suggested [57,58,59,60]. Privacy concerns mean that elderly people prefer to be monitored unobtrusively rather than by camera-based systems [56].

One solution to the privacy issue is to install passive infrared (PIR) sensors in the living environment of the elderly to monitor elderly residents unobtrusively with an ADL classification model [61,62,63,64]. Previous research [65] suggested a new smart radar sensor system that uses an ultra-wideband signal to detect motion. Such radar sensors have a low signal-to-noise ratio and are highly sensitive to environmental changes.

Various indoor activity detection models have been proposed [66,67,68,69,70,71,72,73], most of which use ML to recognize the activities. As stated in [74], deep learning and RNN models have promising results and need to be investigated further for non-intrusive activity recognition. Open datasets from real-life scenarios are used to train and test these models, and the Aruba dataset from the Center for Advanced Studies in Adaptive Systems (CASAS) at Washington State University is often used [75,76].

The authors have published several studies [55,58,77] used CASAS Aruba dataset, where [55] detected travel patterns of a resident living alone using PIR binary sensory data [55]; on the contrary, [58,77] detected the activities of a resident using converted temporal sensory events of each activity samples into an image that is fed into DCNN (Deep Convolutional Neural Networks). First, features are extracted with convolutional layers, and then activity is classified with FCNN (Fully Connected Neural Network).

**Figure 1:**
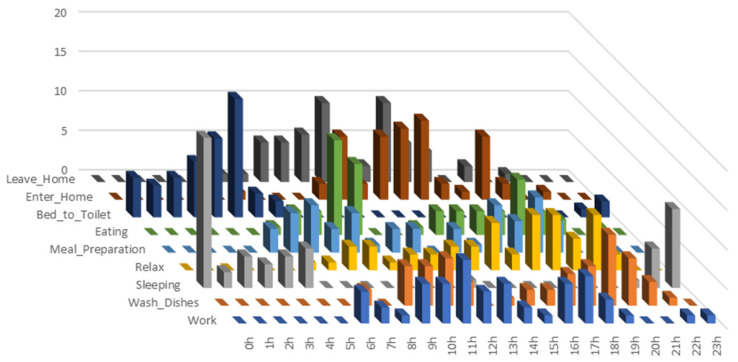
Number of current activity instances in terms of *begin time-stamp*.

The results of the current work proposed in this study outperformed the existing methods on the Aruba dataset [62,72,78,79]. None of the state-of-the-art (SoTA) methods tested on the Aruba dataset for ADL recognition use temporal features, in particular *previous activity* and *begin time-stamp*, which depend significantly on the current activity (see Table 1 and Figure 1).

Herein, we propose a deep-learning model for classifying ADLs from PIR binary sensor data. The model uses a bidirectional long short-term memory (Bi-LSTM), a type of recurrent neural network (RNN) and a fully connected neural network (FCNN) to extract features and classify activities, respectively. The work is not focused on generalizing the model over different houses and for residents with different habits.

The main contributions of this study are as follows:

- Use of the external temporal features, *previous activity* and *begin time-stamp*, that are concatenated with extracted features by the Bi-LSTM before being fed into the FCNN for classification;
- For Bi-LSTM, an input length is empirically determined to be 20 based on the highest accuracy;
- *F*_1_ score difference between the models with/without external features was 28.8%;
- A comparison study on the model’s different architectures, with/without external features and various number of nodes for Bi-LSTM, is conducted;
- For a fair comparison, the proposed model is evaluated on the CASAS Aruba public dataset [76];
- The method outperforms the existing methods [62,72,78,79] with a relatively high *F*_1_ score of 0.917, which is an improvement of 6.25% compared with the existing best *F*_1_ score.

The rest of this paper is organized as follows. Section 2 reviews other activity recognition methods, and Section 3 describes the present method for recognizing ADLs. Section 4 describes how the model was trained, tested, and compared with the other methods. Section 5 discusses the results and Section 6 presents the conclusions.

## 2. Related Works

Previous studies have proposed models that use ML to recognize and classify ADLs from the main data sources for doing so, namely wearable devices and smart homes equipped with depth cameras and binary sensors.

An algorithm [31] was suggested for classifying six ADLs with two inertial measurement units. The algorithm has four stages, that is, filtering, phase detection, posture detection, and activity detection. It detects the body posture during static phases and recognizes types of dynamic activities between postures using a rule-based approach. The model achieves an overall accuracy of 84–97% for different types of activities. However, such methods require intensive handcrafting when other activities are added and are sensitive to distortion of input data. Deep-learning methods are used intensively to extract the features for activity recognition. Bianchi et al. [32] proposed an HAR system based on a wearable IoT sensor, for which the feature extractor was a CNN. The model achieved an accuracy of 92.5% on a standard dataset from the UCI ML Repository. A previous study [45] suggested a model that detects falling and its precursor activity from an open dataset. For classifying falls, the authors employed various methods that are support vector machines (SVMs), random forest, and k-nearest neighbors, which achieve *F*_1_ scores of 0.997, 0.994, and 0.997, respectively, and for classifying the precursor activities, they achieve *F*_1_ scores of 0.756, 0.799, and 0.671, respectively. The results of activity classification are not as good as those of other models. Furthermore, although the systems based on wearable devices provide accurate information about human activity, complications arise like (i) continuing to wear the device, (ii) maintaining battery level, and (iii) attaching the device to the skin.

Systems based on RGB (red–green–blue) cameras [46,47,48] solve the aforementioned problems associated with wearable devices. In a previous study [49], automated ML and transfer learning were used to detect ADLs by analyzing the video from an RGB camera. However, the use of RGB cameras raises privacy concerns.

Activity detection based on depth cameras is another popular method with high precision and less invasion of privacy compared to normal camera images. Anitha et al. [53] proposed an elderly-monitoring system that detects abnormal activities such as falls, chest pain, headache, and vomiting from video sequences with a model based on a dynamic Bayesian network. Image silhouettes are extracted from a normal video sequence that is input to the model, and the model achieves an activity detection accuracy of 82.2%. Jalal et al. [59] developed an HAR model using multiple hidden Markov models that are trained for each specific action. For training and recognizing, the model extracts the features from human depth silhouettes and body-joint information for human activities. The model achieved recognition accuracies of 98.1% and 66.7% on the MSR Action 3D open dataset and a self-annotated dataset, respectively. Hbali et al. [51] presented a method that extracts a human-body skeletal model from depth-camera images, with the classifier being the extremely-randomized-trees algorithm. Although it does not outperform similar models, it provides the promising results with an accuracy of 73.43% on the MSR Daily Activity 3D dataset. Activity recognition systems based on depth cameras are applicable and preferable for the detailed activities such as arm waving or forward kicking, but they do not address the privacy issue fully.

Equipping the living environment of an elderly person with binary sensors invades her/his privacy less than the depth camera does, and it offers greater comfort by avoiding the need to support a wearable device. Yala et al. [61] introduced several traditional ML methods preceded with different feature-extraction techniques, where the highest *F*_1_ score among the experimented methods was 0.662. Machot et al. [78] proposed an activity recognition model that finds the best sensor set for each activity. They used an SVM as the classifier and achieved an *F*_1_ score of 0.82 on the Aruba dataset. Yatbaz et al. [72] suggested two ADL recognition methods based on scanpath trend analysis (STA), one of which gives the highest *F*_1_ score of 0.863 among the existing SoTA models [62,78,79] that are tested on the Aruba dataset. Krishnan et al. [64] proposed a term *previous activity* in their two step activity recognition method.

**Figure 2:**
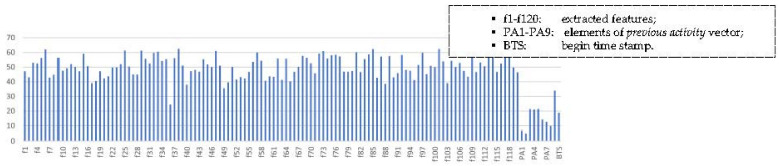
Stacked column graph of absolute values from correlation matrix of features.

However, none of the aforementioned methods, except [64], use temporal features such as *begin time-stamp* and *previous activity*. Figure 2 represents the prominence of external features with stacked column chart, from correlation matrix of feature elements, where vertical axis represents the sum of absolute values of each element for the matrix columns, extracted and external features. External feature elements, with smallest values in the chart, shows that they have less correlation with other features. Our proposed model concatenates these temporal features, *begin time-stamp* and previous value, with the features extracted by the RNN, and it outperforms the existing SoTA models with an *F*_1_ score of 0.917 on the Aruba dataset.

## 3. Methods

The proposed model uses Bi-LSTM, a type of RNN, to extract the feature vectors from an input data sequence, which are then combined with external features including *previous activity* and *begin time-stamp*. The activity recognition is performed by an FCNN. The model structure is empirically selected from extensive experiments over on different combinations of modules (See Section 4.5 and Table 7).

### 3.1. Model Architecture

**Figure 3:**
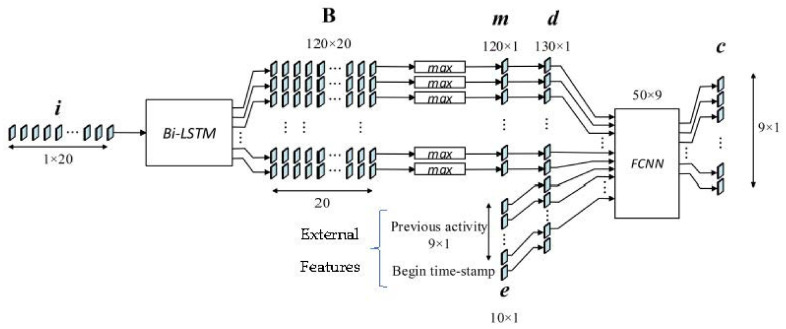
Architecture of the proposed model with its data flow.

Figure 3 shows the architecture of the proposed model, where a pre-processed sequence of sensor data ***i****^T^* = {*i*_0_, *i*_1_, *i*_2_, …, *i*_19_} (see Section 3.3) is inputted to a Bi-LSTM, which consists of an RNN with 60 nodes. Sensors does not send sensor status with consistent frequency. Instead, they send sensor events with “ON” and “OFF” message upon activations. Therefore, the length of the sensor data sequence is inconsistent for activity instances. In order to align data sequence length, zero padding is used in front of the data when the size is less than 20. For sequences longer than 20, the last 20 elements in the sequence form the input data, formulated as follows:

(1) $$i=\left\{\begin{array}{cc} {\left\{z,\;s\right\}}^{T}; & l<20\\ {\left\{s_{l-19},\;\ldots ,s_{l}\right\}}^{T}; & l\geq 20 \end{array}\right.$$

where *s* is the activity sequence with length *l*.

The vector ***z*** represents the zero padding which converts the length of the input sequence to 20 if it was shorter than 20.

**Figure 4:**
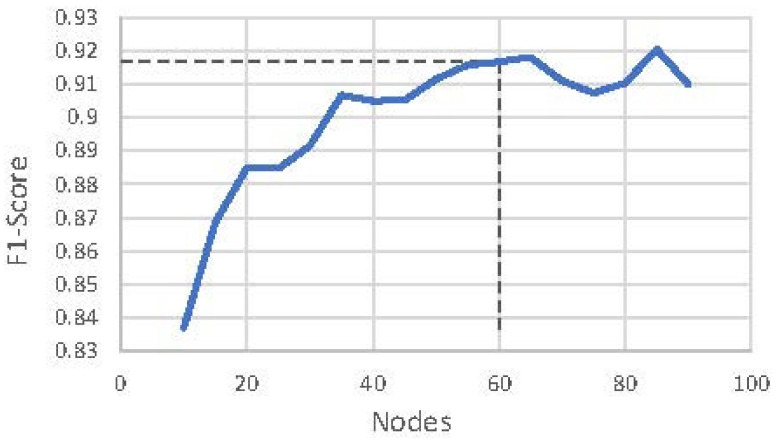
Graph of *F*_1_ score versus Bi-LSTM node.

Empirically, we chose Bi-LSTM over LSTM because of the higher performance, where *F*_1_ score LSTM is 0.842 (See Table 7). Moreover, the number of nodes (60) was chosen empirically at the value where the *F*_1_ score is stabilized (Figure 4). Because the RNN is bidirectional (Bi-LSTM), its output dimensionality (120) is twice its number of nodes (60).

The output vectors of the Bi-LSTM form a matrix ***B***, where each row represents a feature value and each column is a feature vector generated by the Bi-LSTM from the corresponding element of the input sequence. Therefore, the size of matrix ***B*** (120 × 20) is the result of the 60 nodes of the Bi-LSTM and 2 time steps in the input.

On top of this, the feature vector is formed by selecting the maximum value by the max-pooling layer from each row of matrix ***B***. This eliminates the time-step dependency of the features for an activity and selects the maximum value of the feature after taking the whole sequence into account. Each element of the feature vector is selected as

***m_k_*** = **max**(***b_k_*_1_**, ***b_k_*_2_**, ***b_k_*_3_**, … ***b_k_*_20_**) (2)

where *k* is the number of elements in the vector, which is the same as the row number of rows of ***B***.

The external feature vector ***e*** consists of *previous activity **p*** and *begin time-stamp t_s_* [Equation (3)]. Vector ***e*** and the extracted feature vector m are concatenated to form vector ***d*** [Equation (4)], which is then fed into the FCNN classifier:

***e^T^*** = [***p^T^***, *t_s_*], (3)

***d^T^*** = [***m^T^***, ***e^T^***]. (4)

*Previous activity****p*** is given in 9 × 1 one-hot vector form where each element represents an activity. *Begin time-stamp t_s_*, the beginning hour of the activity, corresponds to the current activity, whereas current activity is known to be dependent on *previous activity*. Table 1 tabulates the number of *previous activities’* instances with respect to the current activity in the balanced dataset, where the values present a clear association between the current activity and those that preceded it; for example, Sleeping happens mostly before Bed_to_Toilet. Moreover, the stacked column chart of absolute values (Figure 2) from the correlation matrix for all features, including extracted and external, reveals that *previous activity* vector elements and *begin time stamp* are less correlated to other features.

The number of instances of *begin time-stamp* in terms of daily hours is represented in Figure 1, where the three-dimensional graph exposes the associations between activities and their starting time interval.

The FCNN executes the classification and consists of a 50-node hidden layer and a nine-node output layer. Activation functions of the hidden and output layers are ReLu and Sigmoid, respectively. The input vector ***d*** consists of three elements [Equations (3) and (4)]: the feature vector ***m***, the *previous activity* ***p***, and the *begin time-stamp t_s_*. The fully connected classification network is defined as

(5) $$a^{h}=ReLu\;\left(W^{h}d+b^{h}\right)$$

(6) $$a^{o}=\sigma \;\left(W^{o}a+b^{o}\right)$$

where ***a****^h^* and ***a****^o^* are the outputs of the hidden and output layers, respectively, of the network.

### 3.2. Dataset

**Figure 5:**
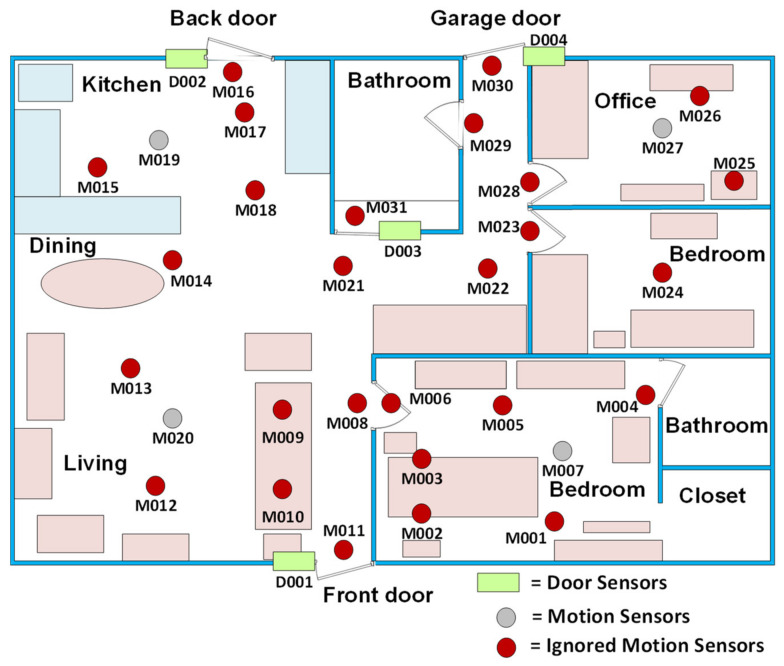
Aruba testbed layout where motion and door sensors are depicted.

We used Aruba open data set from CASAS smart home project [76] to train and evaluate our model. CASAS assembled 64 open datasets from equipped smart houses inhabited by single or multiple residents for certain amounts of time. Its inhabitancy duration and frequent use in model evaluations led us to use the Aruba testbed dataset in the present work. As shown in Figure 5, Aruba is a smart house in which an elderly lady lived alone for seven months. This house is equipped with 31 wireless binary motion sensors, four temperature sensors, and four door sensors. Because we used only motion sensor data, from all 31 motion sensors and four door sensors, the temperature sensors are not depicted in Figure 5 [77].

**Figure 6:**
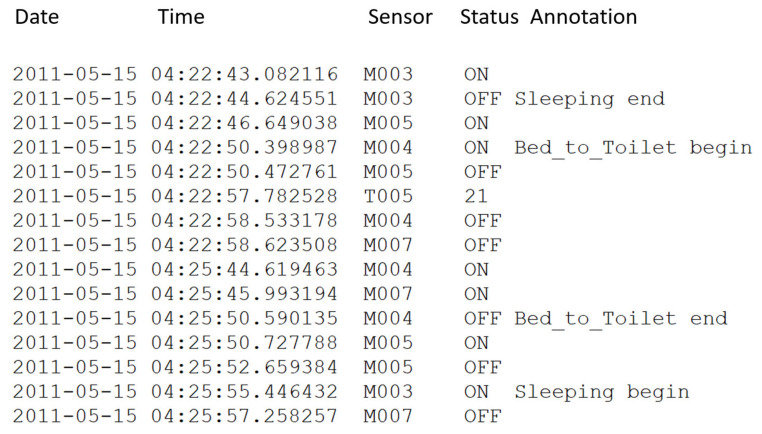
Format of Aruba dataset.

The open dataset is formatted as shown in Figure 6, where each instance consists of date, time, sensor status, and annotations. The dataset is a list of actions lasting for 219 days from 4 November 2010 to 6 November 2011, and it comprises 1′719′557 registered events in total. Figure 6 lists the sensor instances of two actions, namely Sleeping and Bed_to_Toilet, which happened during the night of 15 May 2011. Here, the activity Bed_to_Toilet happens between two Sleeping activities, which is intuitive.

### 3.3. Preprocessing of Dataset

For a fair comparison, we used the same data preprocessing method as the one described in [72]. The Aruba dataset contains 1′719′557 raw sensor samples in total. First, we removed all irrelevant samples, i.e., temperature sensor samples, from the dataset, leaving 1′602′981 samples. Moreover, for the sake of formatting, the door sensor statuses of “OPEN” and “CLOSE” were replaced with “ON” and “OFF”, respectively. Various incorrect labels of the sensor status “OFF” (e.g., “OF” and “OFF5”) were replaced with “OFF”. After these steps, the external features, i.e., *previous activity* and *begin time stamp*, of each activity, are extracted from the dataset and merged with sensory data of each activity instances. We employed 10-fold cross-validation to evaluate the proposed method, ignoring the activities of Housekeeping and Respirate because they had only 33 and six samples, respectively. The Aruba dataset is imbalanced in terms of the number of samples for each class, ranging from six to 2919 as shown in Table 2. To balance the dataset, 60 samples were selected randomly from each class; thus, six samples were allocated for each fold. Therefore, for each fold evaluation, 90% (54 samples) and 10% (six samples) of the particular class samples were used as the training set and the testing set, respectively.

**Figure 7:**
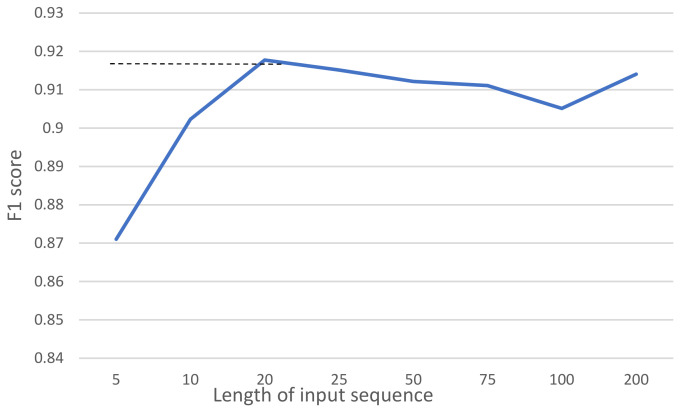
Sample distribution in terms of sensor events.

Figure 7 represents a *F*_1_ scores vs. input data length graph. Empirically, the highest *F*_1_ score of 0.917 is given against input data length of 20. Therefore we set the input data length as 20 for reducing the computational complexity. In case of the data length of an activity which is less than 20 zero padding is used to fit the sequence to the model input.

### 3.4. Evaluation Measures

Via one of the most commonly used model-validation techniques, we used stratified 10-fold cross-validation to assess our model. For a fair comparison, we selected 60 samples randomly from each activity sample set, resulting in 540 random samples in total. The selected sample set was partitioned into two subsets, namely the training set and the testing set, with 90% and 10%, respectively, of the samples of each activity. Therefore, 54 and six samples were allocated for train and test sets, respectively.

The proposed model was evaluated in terms of the following measures: Recall, Precision, *F*_1_ score, Specificity, Accuracy, and Error. These measures were calculated from the model’s numbers of true and false prediction: TP (true positive), TN (true negative), FP (false positive), and FN (false negative) [62]. Evaluation scores of the model are averaged scores from the results of five different models trained and tested on five different sample sets.

### 3.5. Technical Specifications

Model training was performed on a DGX1 supercomputer, whereas the testing was performed on an ordinary server computer. The server computer was a Dell Workstation 7910 with a six-core Intel(R) Xeon(R) CPU E5-2603 v3 @ 1.60 GHz, 16 GB RAM, and GTX Titan X GPU.

## 4. Results

We used stratified 10-fold-cross validation for evaluation of the models. Each row of Table 3, Table 4 and Table 7 represent the weighted average of 10-fold evaluation results of five different models that are trained on five different datasets.

### 4.1. Activity Recognition with Extra Features

Table 3 presents the results for the model with the external features of *previous activity* and *begin time-stamp*. The normalized values of the confusion matrix for the activities are presented on the left side of the table, while the performance measures of Precision, Recall, Specificity, *F*_1_ score, Accuracy, and Error are presented on the right side. The best performance of the model was achieved for the Enter_Home activity, where its Precision, Recall, Specificity, *F*_1_ score and Accuracy were 0.997, 1.000, 1.000, 0.978, and 99.96%, respectively. The second and third-best performances were for the Bed_to_Toilet and Leave_Home activities, with *F*_1_ scores of 0.990 and 0.987, respectively. The worst-recognized activities were Meal_Preparation and Wash_Dishes, with *F*_1_ scores of 0.824 and 0.821, respectively. The *F*_1_ score of Wash_Dishes was 17.6% lower than that of the best-recognized activity (Enter_Home).

### 4.2. Activity Recognition without Previous Activity Feature

Table 4 tabulates the results for the model without accounting for the external feature of *previous activity*. The best-recognized activity was Bed_to_Toilet, where its Precision, Recall, Specificity, *F*_1_ score, and Accuracy were 0.907, 0.973, 0.988, 0.939, and 98.59%, respectively. The second-best-recognized activity was Sleeping, where its Precision, Recall, Specificity, *F*_1_ score, and Accuracy were 0.952, 0.793, 0.995, 0.865, and 97.26%, respectively. The worst-recognized activity was Leave_Home, with an *F*_1_ score of 0.526, which was 44% lower than the highest *F*_1_ score of the Bed_to_Toilet activity.

Furthermore, the best *F*_1_ score of this model was 5.9% lower than the highest *F*_1_ score of the model with the *previous activity* feature (Table 3). On the other hand, the lowest *F*_1_ score of this model was 35.9% lower than the worst *F*_1_ score of the model with the *previous activity*.

### 4.3. Classification Results on the Remaining Dataset

The confusion matrix and performance measure Recall on the remaining dataset are represented in Table 5. The best-recognized activities are Enter_Home, Bed_to_Toilet and Leave_Home with Recall of 0.998, 0.997 and 0.990, respectively. The worst-recognized activities are Meal_Preparation and Wash_Dishes with a Recall measure of 0.825 and 0.860, respectively. The worst-recognized activity Meal_Preparation’s performance measure Recall is 17.3% lower than the best-performer’s result. The overall average of performance measure Recall on the remaining dataset is 0.923.

### 4.4. Real-Time Activity Recognition with a Predicted Previous Activity Feature

For all the previously mentioned experiments, the *previous activity* feature was a ground truth which is extracted from the dataset. Table 6 represents weekly-basis real-life scenario activity recognition results on the whole 6 months dataset where the *previous activity* feature were predicted (not the ground truth) by the proposed method. For the sake of simplicity, we chose to report the weekly-basis results as the daily-basis results were similar. We used the very first activity, the ground truth, of the week where the start of the week was set with Sleeping activity, as a previous activity feature for the second activity. After predicting the second activity, the predictions are used as a *previous activity* feature for its next activity. In Table 6, a confusion matrix and Recall measure (Recall is chosen as the dataset is severely imbalanced) of the test result is tabulated. The best recognized activities are Work, Leave_Home and Enter_Home with Recall of 0.977, 0.972 and 0.970, respectively. The worst recognized activities are Meal_Preparation and Eating, with a Recall of 0.714 and 0.850, respectively.

For a comparison, the best Recall measure of the model with predicted *previous activity* feature is 2.14% lower than the Recall measure of the model with the ground truth *previous activity* feature (Table 5) while the worst recognized activity of the model with the predicted *previous activity* feature is 15.54% lower than the Recall measure of the worst recognized activity of the model with ground truth *previous activity* feature (Table 5).

### 4.5. Comparison Study of Proposed Model with Different Combinations of Internal Modules

Table 7 represents the 10-fold cross-validation results of a comparative study of the proposed model with and without external features for unidirectional and bidirectional LSTM RNNs with different numbers of nodes. The best *F*_1_ score is 0.917 for model 3, which has a Bi-LSTM with 60 nodes as a feature extractor and two external features, *previous activity* and *begin time-stamp*. The second-best *F*_1_ score was 0.905 for model 4, which consists of external features and a Bi-LSTM with 50 nodes. Furthermore, the third-best *F*_1_ score was 0.892 for model 7, which has only one external feature (*previous activity*) and a Bi-LSTM with 60 nodes. The worst *F*_1_ score was 0.495 for model 2, which has a 60-node unidirectional LSTM feature extractor but no external features.

### 4.6. Training vs. Testing Accuracy

**Figure 8:**
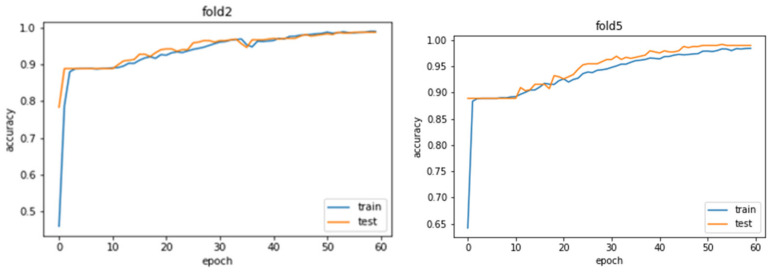
Train and Test accuracy.

For training and testing the model, the 10-fold cross validation is used (See Section 3.2, Section 3.3 and Section 3.4). Figure 8 represent train and test graphs for accuracy and loss function after 60 epochs. Train and test curves converge and final values exceed 0.9 for accuracy and is lower than 0.1 for a loss function.

### 4.7. Classification Latency

The average classification latency of the model for each activity is 26.4 ms, and the maximum latency is 30.3 ms, as represented in Table 8. Because the classification latency is less than 30 ms, which is within the industrial IoT system latency requirement of 100 ms [80], the model can be used in an IoT-based real-time privacy-preserving ADL recognition system. The latency measurement was performed on a server with moderate specifications (Section 3.5).

### 4.8. Comparison with SoTA Models

Table 9 (fair) and Table 10 (non-fair) compare the proposed model with existing SoTA models: [62] by Gochoo et al. [72] by Yatbaz et al. [78,79] by Machot et al. that were evaluated on CASAS Aruba dataset. Table 9 represents a fair comparison with two models [62,72] which are evaluated with the same technique in their studies (Section 3.4). The proposed model outperformed these two SoTA models in a fair comparison with an *F*_1_ score of 0.917, which is 6.26% and 16% higher than those of the second-best-performing model STA [72] and our previous study [62], respectively (Table 9).

Machot et al. [78,79] respectively employed an SVM and RNN as a classifier and they are evaluated on a CASAS Aruba dataset. While both studies [78,79], used 10-fold cross validation technique to evaluate their classification models, [78] used imbalanced dataset with classification penalty and [79] used Synthetic Minority oversampling technique [81] to make the dataset balanced. Table 10 represents comparison of our model achieving an *F*_1_ score that is 11.8% and 7.88% higher than those of the models in [78,79]. Although [72], the latest and highest performer of classification task on CASAS Aruba dataset claims that they outperformed these models of [78,79], moreover, due to the difference of sampling methods used in [78,79] for preparing datasets, Table 10 is not a fair comparison.

## 5. Discussion

Concatenating the external features of *previous activity* and *begin time-stamp* to the extracted features from Bi-LSTM gives remarkable results on classifying ADLs from binary sensor data. Our model outperforms all the SoTA models on the Aruba testbed dataset, where its *F*_1_ scores range between 0.821 and 0.998 with an average of 0.917.

The model predicts some activities much better than do other models, such as Meal_Preparation, Eating, Wash_Dishes, Enter_Home, and Leave_Home, because of adding extra features, especially *previous activity*. For example, the Enter_Home activity is classified with 99.96% accuracy (Table 3) because it always occurs after Leave_Home.

We chose to have 60 nodes in the Bi-LSTM as a trade-off between computational complexity and accuracy (see Figure 4). As it can be seen from Table 7, the *F*_1_ score of the model decreases to 0.905 and 0.855 when only 50 and 20 nodes are respectively used.

Training and testing accuracy and loss graphs represent good fit of the model by converging and having high values greater than 0.9 for accuracy and low values, less than 0.1, for loss function (Figure 8).

The worst activity recognition of our model is on Wash_Dishes with an *F*_1_ score of 0.821; nevertheless, this is 0.12% and 3.92% higher than the average *F*_1_ scores of existing models [62,78], respectively.

The model is tested on remaining dataset which was not part of the balanced dataset to train and test the model. Instead of taking *F*_1_ score as the main measure, the performance measure Recall represents reasonable results since the remaining part of the dataset is imbalanced.

The model is evaluated with a predicted (not the ground truth) *previous activity* feature on weekly activity sequences to simulate a real-life scenario activity recognition (Table 6), as well. The results show a reasonably high Recall of 0.927. However, as expected, the best and worst Recall measures were degraded down 2.14% and 15.54%, respectively, compared to the model performance with ground truth *previous activity* feature (Table 5).

As well as outperforming the SoTA models, the classification latency of the proposed model is less than 30 ms, which is fast enough for an IoT-based real-time privacy-preserving activity recognition system.

The model’s worst performance is on classifying between the activities of Meal_Preparation and Wash_Dishes due to the two actions occurring in the same location in the house, namely the kitchen. This classification could be improved by placing other sensors (e.g., temperature, humidity) in the kitchen.

Although the external feature *begin time-stamp* improved the performance of the model, its contribution to the *F*_1_ score was not as great as that of the external feature *previous activity* (Table 7). Despite the fact that the external feature *previous activity* has prominent effect on the model performance, it might mislead to misclassification when wrong *previous activity* is generated automatically from previous step of classification.

Due to lack of similar datasets, our model is trained and tested only on CASAS dataset. If the model is employed to classify daily activities of a new resident, it is necessary for the model to have a learning phase in order to capture the resident’s daily activity pattern.

## 6. Conclusions and Future Works

We proposed a privacy-preserving activity recognition model concatenating Bi-LSTM extracted features and external temporal features for classifying the ADLs of an elderly person living alone. The dataset used to train and test the model was the CASAS Aruba open dataset, which was collected from binary sensors in a smart home in which an elderly resident lived alone for seven months. The model outperformed the existing SoTA models with the highest *F*_1_ score of 0.917, which was 6.26% better than that of the best existing model. Moreover, a classification latency of less than 30 ms allows our model to be placed in a server of an IoT-based ADL recognition system.

For future work, the worst activity classifications, namely those of Meal_Preparation and Wash_Dishes, both of which take place in the kitchen, should be improved by adding other sensors such as a thermometer, a humidity meter, and an ammeter for electrical appliance. Moreover, a multi-resident activity recognition model should be developed for elderly-monitoring IoT systems.

## Figures and Tables

**Table 1:** Number of *previous activity* instances for activities selected in train and test sets.

	Previous Activity	Work	Wash_Dishes	Sleeping	Relax	Meal_Preparation	Eating	Bed_to_Toilet	Enter_Home	Leave_Home
Activity										
Work		10	2	0	29	4	1	0	8	0
Wash_Dishes		1	1	0	19	2	31	0	0	0
Sleeping		1	0	3	35	0	0	15	0	0
Relax		1	2	0	25	21	1	0	4	0
Meal_Preparation		1	0	13	15	19	1	0	5	0
Eating		0	0	0	2	43	8	0	1	0
Bed_to_Toilet		0	0	54	0	0	0	0	0	0
Enter_Home		0	0	0	0	0	0	0	0	54
Leave_Home		0	1	0	24	7	8	0	14	0

**Table 2:** Number of Instances for Activities.

	Activities	Number of Instances
1	Meal_Preparation	1606
2	Relax	2919
3	Eating	257
4	Work	171
5	Sleeping	401
6	Wash_Dishes	65
7	Bed_to_Toilet	157
8	Enter_Home	431
9	Leave_Home	431
10	Housekeeping	33
11	Respirate	6

**Table 3:** Confusion Matrix for Activities and Evaluation of Model with Previous Activity Feature.

	Prediction	Work	Wash_Dishes	Sleeping	Relax	Meal_Preparation	Eating	Bed_to_Toilet	Enter_Home	Leave_Home	Precision	Recall	Specificity	*F*_1_ Score	Accuracy	Error
Activity																
Work		0.913	0.007	0.040	0.010	0.000	0.027	0.000	0.000	0.003	0.881	0.913	0.985	0.897	97.667	2.333
Wash_Dishes		0.010	0.833	0.000	0.013	0.133	0.010	0.000	0.000	0.000	0.809	0.833	0.975	0.821	95.963	4.037
Sleeping		0.047	0.000	0.937	0.010	0.000	0.003	0.003	0.000	0.000	0.934	0.937	0.992	0.935	98.556	1.444
Relax		0.023	0.003	0.010	0.900	0.013	0.047	0.000	0.000	0.003	0.918	0.900	0.990	0.909	98.000	2.000
Meal_Preparation		0.003	0.173	0.003	0.000	0.817	0.003	0.000	0.000	0.000	0.831	0.817	0.979	0.824	96.111	3.889
Eating		0.040	0.010	0.010	0.047	0.020	0.873	0.000	0.000	0.000	0.907	0.873	0.989	0.890	97.593	2.407
Bed_to_Toilet		0.000	0.000	0.003	0.000	0.000	0.000	0.990	0.000	0.007	0.990	0.990	0.999	0.990	99.778	0.222
Enter_Home		0.000	0.000	0.000	0.000	0.000	0.000	0.000	1.000	0.000	0.997	1.000	1.000	0.998	99.963	0.037
Leave_Home		0.000	0.003	0.000	0.000	0.000	0.000	0.007	0.003	0.987	0.987	0.987	0.998	0.987	99.704	0.296
Mean value (all)											**0.917**	**0.917**	**0.990**	**0.917**	**98.148**	**1.852**

**Table 4:** Confusion Matrix for Activities and Evaluation of model without Previous Activity Feature.

	Prediction	Work	Wash_Dishes	Sleeping	Relax	Meal_Preparation	Eating	Bed_to_Toilet	Enter_Home	Leave_Home	Precision	Recall	Specificity	*F*_1_ score	Accuracy	Error
Activity																
Work		0.870	0.000	0.02	0.037	0.000	0.073	0.000	0.000	0.000	0.759	0.870	0.965	0.811	95.481	4.519
Wash_Dishes		0.007	0.617	0.000	0.003	0.360	0.013	0.000	0.000	0.000	0.613	0.617	0.951	0.615	91.407	8.593
Sleeping		0.143	0.000	0.793	0.033	0.000	0.027	0.003	0.000	0.000	0.952	0.793	0.995	0.865	97.259	2.741
Relax		0.073	0.017	0.003	0.827	0.01	0.06	0.007	0.000	0.003	0.808	0.827	0.975	0.817	95.889	4.111
Meal_Preparation		0.007	0.343	0.000	0.000	0.630	0.02	0.000	0.000	0.000	0.618	0.630	0.951	0.624	91.556	8.444
Eating		0.037	0.023	0.01	0.123	0.02	0.787	0.000	0.000	0.000	0.803	0.787	0.976	0.795	95.481	4.519
Bed_to_Toilet		0.000	0.000	0.003	0.000	0.000	0.000	0.973	0.02	0.003	0.907	0.973	0.988	0.939	98.593	1.407
Enter_Home		0.007	0.003	0.003	0.000	0.000	0.000	0.023	0.653	0.310	0.578	0.653	0.940	0.613	90.852	9.148
Leave_Home		0.003	0.003	0.000	0.000	0.000	0.000	0.067	0.457	0.470	0.597	0.470	0.960	0.526	90.593	9.407
Mean value (all)											**0.737**	**0.736**	**0.967**	**0.734**	**94.123**	**5.877**

**Table 5:** Confusion Matrix for Remaining Activities and Recall Measure.

	Prediction	Work	Wash_Dishes	Sleeping	Relax	Meal_Preparation	Eating	Bed_to_Toilet	Enter_Home	Leave_Home	Recall
Activity											
Work		0.880	0.000	0.003	0.034	0.000	0.082	0.000	0.000	0.001	0.880
Wash_Dishes		0.000	0.860	0.000	0.008	0.132	0.000	0.000	0.000	0.000	0.860
Sleeping		0.040	0.000	0.946	0.006	0.001	0.007	0.001	0.000	0.000	0.946
Relax		0.053	0.005	0.006	0.915	0.009	0.008	0.000	0.000	0.004	0.915
Meal_Preparation		0.001	0.161	0.000	0.005	0.825	0.008	0.000	0.000	0.000	0.825
Eating		0.021	0.008	0.000	0.045	0.029	0.896	0.000	0.000	0.000	0.896
Bed_to_Toilet		0.000	0.000	0.001	0.000	0.000	0.000	0.997	0.000	0.001	0.997
Enter_Home		0.000	0.000	0.000	0.000	0.001	0.000	0.000	0.998	0.000	0.998
Leave_Home		0.002	0.002	0.001	0.002	0.000	0.001	0.003	0.000	0.990	0.990
Mean value (all)											**0.923**

**Table 6:** Confusion Matrix for Activities and Evaluation of model with Predicted Previous Activity Feature.

	Prediction	Work	Wash_Dishes	Sleeping	Relax	Meal_Preparation	Eating	Bed_to_Toilet	Enter_Home	Leave_Home	Recall
Activity											
Work		0.977	0.000	0.000	0.000	0.000	0.023	0.000	0.000	0.000	0.977
Wash_Dishes		0.000	0.969	0.000	0.000	0.031	0.000	0.000	0.000	0.000	0.969
Sleeping		0.030	0.000	0.965	0.003	0.003	0.000	0.000	0.000	0.000	0.965
Relax		0.018	0.007	0.007	0.958	0.008	0.002	0.000	0.000	0.000	0.958
Meal_Preparation		0.001	0.282	0.000	0.001	0.714	0.003	0.000	0.000	0.000	0.714
Eating		0.000	0.041	0.000	0.065	0.045	0.850	0.000	0.000	0.000	0.850
Bed_to_Toilet		0.000	0.000	0.000	0.007	0.000	0.000	0.966	0.000	0.028	0.966
Enter_Home		0.000	0.000	0.000	0.002	0.000	0.000	0.019	0.970	0.009	0.970
Leave_Home		0.000	0.002	0.000	0.000	0.000	0.002	0.019	0.005	0.972	0.972
Mean value (all)											**0.927**

**Table 7:** Comparison Results of Proposed Model with Different Combinations of Modules.

Model	Previous Activity	Begin Timestamp	No. of LSTM Nodes	No. of Bi-LSTM Nodes	Precision	Recall	Specificity	*F*_1_ Score	Accuracy	Error
1	✓	✓	60	-	0.844	0.843	0.980	0.842	96.510	3.490
2	-	-	60	-	0.495	0.484	0.935	0.474	88.527	11.473
3	**✓**	**✓**	**-**	**60**	**0.917**	**0.917**	**0.990**	**0.917**	**98.148**	**1.852**
4	✓	✓	-	50	0.906	0.905	0.988	0.905	97.893	2.107
5	✓	✓	-	20	0.886	0.885	0.986	0.885	97.440	2.560
6	-	✓	-	60	0.737	0.736	0.967	0.734	94.123	5.877
7	✓	-	-	60	0.893	0.891	0.986	0.892	97.588	2.412
8	-	-	-	60	0.711	0.714	0.964	0.712	93.654	6.346

**Table 8:** Maximum Classification Latency for Each Activity.

	Activity	Classification Time [s]
1	Meal_Preparation	0.0303
2	Relax	0.0295
3	Eating	0.0257
4	Work	0.0275
5	Sleeping	0.0257
6	Wash_Dishes	0.0249
7	Bed_to_Toilet	0.0246
8	Enter_Home	0.0250
9	Leave_Home	0.0247
10	Average	0.0264
11	Absolute maximum	0.0303

**Table 9:** Comparing Present Model with Existing Models.

No.	Model	Year	*F*_1_ Score
1	DCNN Gochoo et al. [62]	2018	0.79
2	STA Method 2 Yatbaz et al. [72]	2019	0.863
3	**Our model**	**Proposed**	**0.917**

**Table 10:** Non-fair Comparing Present Model with Existing Models.

No.	Model	Year	*F*_1_ Score
1	P-SVM Machot et al. [77]	2017	0.82
2	RNN model Machot et al. [78]	2018	0.85
3	**Our model**	**Proposed**	**0.917**

## Data Availability

The Aruba dataset is provided by Center for Advanced Studies in Adaptive System (CASAS) in School of Electrical Engineering and Computer Science of Washington State University (http://casas.wsu.edu/datasets/). The accessed date to the dataset is 11 July 2021.
